# Cognitive symptoms progress with limbic-predominant age-related TDP-43 encephalopathy stage and co-occurrence with Alzheimer disease

**DOI:** 10.1093/jnen/nlad098

**Published:** 2023-11-15

**Authors:** Satomi Hiya, Carolina Maldonado-Díaz, Jamie M Walker, Timothy E Richardson

**Affiliations:** Department of Pathology, Molecular and Cell-Based Medicine, Icahn School of Medicine at Mount Sinai, New York, New York, USA; Department of Pathology, Molecular and Cell-Based Medicine, Icahn School of Medicine at Mount Sinai, New York, New York, USA; Department of Pathology, Molecular and Cell-Based Medicine, Icahn School of Medicine at Mount Sinai, New York, New York, USA; Nash Family Department of Neuroscience, Icahn School of Medicine at Mount Sinai, New York, New York, USA; Neuropathology Brain Bank & Research CoRE, Icahn School of Medicine at Mount Sinai, New York, New York, USA; Department of Pathology, Molecular and Cell-Based Medicine, Icahn School of Medicine at Mount Sinai, New York, New York, USA

**Keywords:** Alzheimer disease neuropathologic change, Clinical dementia rating, Lewy body dementia, Limbic-predominant age-related TDP-43 encephalopathy neuropathologic change, Mini-Mental State Examination, Primary age-related tauopathy

## Abstract

Limbic-predominant age-related TDP-43 encephalopathy neuropathologic change (LATE-NC) is a neuropathologic entity characterized by transactive response DNA-binding protein of 43-kDa (TDP-43)-immunoreactive inclusions that originate in the amygdala and then progress to the hippocampi and middle frontal gyrus. LATE-NC may mimic Alzheimer disease clinically and often co-occurs with Alzheimer disease neuropathologic change (ADNC). This report focuses on the cognitive effects of isolated and concomitant LATE-NC and ADNC. Cognitive/neuropsychological, neuropathologic, genetic, and demographic variables were analyzed in 28 control, 31 isolated LATE-NC, 244 isolated ADNC, and 172 concurrent LATE-NC/ADNC subjects from the National Alzheimer’s Coordinating Center. Cases with LATE-NC and ADNC were significantly older than controls; cases with ADNC had a significantly higher proportion of cases with at least one *APOE* ε4 allele. Both LATE-NC and ADNC exhibited deleterious effects on overall cognition proportional to their neuropathological stages; concurrent LATE-NC/ADNC exhibited the worst overall cognitive effect. Multivariate logistic regression analysis determined an independent risk of cognitive impairment for progressive LATE-NC stages (OR 1.66; p = 0.0256) and ADNC levels (OR 3.41; p < 0.0001). These data add to the existing knowledge on the clinical consequences of LATE-NC pathology and the growing literature on the effects of multiple concurrent neurodegenerative pathologies.

## INTRODUCTION

Limbic-predominant age-related TDP-43 encephalopathy neuropathologic change (LATE-NC) is a neuropathologic entity thought to be separate from other TDP-43-related proteinopathies, with recently described consensus criteria (1). Previously termed “hippocampal sclerosis of aging,” “hippocampal sclerosis dementia,” and “cerebral age-related TDP-43 and sclerosis” (2), LATE-NC is characterized by transactive response (TAR) DNA-binding protein of 43-kDa (TDP-43)-immunoreactive neuronal cytoplasmic inclusions (3), which originate in the amygdala (Stage 1) and progress to the hippocampus (Stage 2) and middle frontal gyrus of the neocortex (Stage 3) (1, 4). There remains disagreement over this diagnosis and its relationship to other disorders with TDP-43 inclusions, including frontotemporal lobe degeneration with TDP-43 (FTLD-TDP) and amyotrophic lateral sclerosis (ALS). Although there are clinical, genetic, and morphological differences, there indeed appears to be some degree of overlap among these entities (particularly at higher stages) and the distinction between them may be subtle (3, 5–8).

LATE-NC is a frequent finding in autopsy cohorts of elderly subjects and frequently co-occurs with other neurodegenerative disorders, including Alzheimer disease neuropathologic change (ADNC), primary age-related tauopathy (PART), and Lewy body disease (LBD) but it can also be found in patients with minimal or no other evident neurodegenerative pathology (9–14). Clinically, LATE-NC may present similarly to Alzheimer disease, with effects on global cognition, episodic memory, executive function, and language, as well as an increased likelihood of experiencing neuropsychiatric symptoms and extrapyramidal signs (14–21). Given the frequency with which LATE-NC is concurrent with other neurodegenerative disorders, it is difficult to disentangle the cognitive effects of LATE-NC from these other comorbidities. Hence, several recent studies have focused on the cumulative effects of these multiple coexistent neurodegenerative findings (11, 16, 22–24).

In this report, we utilize the National Alzheimer’s Coordinating Center (NACC) database to compare the demographic, genetic, neuropathologic, cognitive, and neuropsychological features in subjects with autopsy-confirmed isolated LATE-NC (n = 31), isolated ADNC (n = 244), concurrent LATE-NC and ADNC (n = 172), and a group of control subjects without significant ADNC, LATE-NC, LBD, FTLD, or other identified neurodegenerative pathologies (n = 28). We demonstrate a progressive deleterious effect of isolated LATE-NC on cognitive function and a subset of neuropsychological domains as TDP-43 immunoreactive inclusions progress from the amygdala to the neocortex (Stages 1–3). This is similar to, (although less severe than), the effects of the progression of ADNC from “low” to “high” level, which consistently affects all cognitive and neuropsychological domains (25, 26). LATE-NC stage and ADNC level were both independently associated with cognitive impairment in multivariate analysis of this cohort. In sum, these results indicate that the presence of LATE-NC has less severe effects on cognition compared to ADNC, and confirm previous literature suggesting that comorbid ADNC and LATE-NC have compounding effects on cognition when they are present together.

## MATERIALS AND METHODS

### Case selection and exclusion criteria

For this study, we downloaded data from the National Alzheimer’s Coordinating Center (NACC), established with funding from the National Institute on Aging (U01 AG016976) (https://naccdata.org/). Standardized Uniform Data Set (UDS), version 3 variable definitions (https://naccdata.org/data-collection/forms-documentation/uds-3), Neuropathology (NP) Data Set, version 11 variable definitions (https://naccdata.org/data-collection/forms-documentation/np-11), and Genetic Data Set (Gen) variable definitions (https://files.alz.washington.edu/documentation/rdd-genetic-data.pdf) from NACC were used, as previously described (27–29). A total of 7709 unique NACC cases where at minimum global clinical dementia rating (CDR) was assessed and the last patient encounter occurred within the final 2 years of life were identified, as previously described (11, 22, 29). Six thousand six hundred twenty cases were excluded for not having sufficient pathologic data available to determine ADNC level, LATE-NC stage, LBD stage, or vascular pathology (Supplementary DataFig. S1). Of the 1089 remaining cases, an additional 614 cases were excluded for having significant comorbid neurodegenerative pathologies, including diagnoses of FTLD, ALS/motor neuron disease, LBD, multiple system atrophy, prion disease, trinucleotide repeat diseases, chronic traumatic encephalopathy, and Down syndrome, leaving 475 cases for analysis (11, 22).

### Neuropathologic, genetic, and demographic variables

LATE-NC stage was assessed at the originating institutions using a combination of phospho-specific and nonphospho-specific antibodies and were not directly rereviewed for this study. For TDP-43 staging, we used the NACC NP dataset variables NPTDPA (TDP-43 immunoreactive inclusions in the spinal cord), NPTDPB (TDP-43 immunoreactive inclusions in amygdala), NPTDPC (TDP-43 immunoreactive inclusions in the hippocampus), and NPTDPE (TDP-43 immunoreactive inclusions in neocortex [middle frontal gyrus]). Cases were assigned LATE-NC Stage 0 in the absence of TDP-43 immunoreactivity in any assessed region, LATE-NC Stage 1 with TDP-43 immunoreactive inclusions in the amygdala only, LATE-NC Stage 2 with TDP-43 immunoreactive inclusions in the amygdala and hippocampus, and LATE-NC Stage 3 with TDP-43 inclusions in the amygdala, hippocampus, and neocortex (1, 8, 30). Cases with TDP-43 immunoreactive lesions in the neocortex but documented diagnoses of FTLD-TDP and/or ALS were excluded, per the most current recommendations for the diagnosis of LATE-NC (8). “Isolated” LATE-NC was defined as cases with LATE-NC which lacked “intermediate”/“high” ADNC, limbic/neocortical LBD, or any additional neurodegenerative proteinopathy.

Where available, ADNC level was determined from the NACC NP dataset variable NPADNC. In instances where NPADNC was not available, ADNC levels were derived from a combination of Braak stage (NACCBRAA), Thal phase (NPTHAL), and CERAD neuritic plaque (NP) score (NACCNEUR) (25, 31–33). Cases without sufficient data on Braak stage, Thal phase, and CERAD NP score to determine a specific ADNC level were excluded, except in the group with concurrent LATE-NC Stage 2–3 and ADNC Level 2–3, where we included cases that were missing Thal phase but still could be placed in either “intermediate” or “high” level of ADNC (Level 2–3) based on Braak stage and CERAD NP scores alone (25). Hippocampal sclerosis was assessed with the NP variable NPHIPSCL. In total, 28 cases were included as controls (those lacking any ADNC, LATE-NC, LBD, FTLD, or other significant neuropathologic diagnoses), 31 cases were included as isolated LATE-NC (5 LATE-NC Stage 1, 19 LATE-NC Stage 2, and 7 LATE-NC Stage 3), 244 cases were included as isolated ADNC (17 ADNC Level 1, 107 ADNC Level 2, and 120 ADNC Level 3), and 172 were included as concurrent LATE-NC/ADNC (4 with LATE-NC Stage 1/ADNC Level 1 and 168 with LATE-NC Stages 2–3/ADNC Levels 2–3).

Patient age was derived from the UDS variable for age at death NACCDAGE. Gender was assessed with the UDS variable SEX and education was assessed with the UDS variable EDUC. *APOE* genotype (ε2/2, ε2/3, ε2/4, ε3/3, ε3/4, ε4/4) were assessed with the variable NACCAPOE. Patient race was assessed with the UDS variable NACCNINR, and included White/Caucasian, Black/African American, Asian, American Indian/Alaska Native, and Native Hawaiian/Pacific Islander. Demographic data on all individuals included in this study are shown in the Table.

### Cognitive and neuropsychological variables

Representative cognitive and neuropsychological variables encompassing overall cognition and specific neuropsychological domains were assessed using the UDS variable definitions, from the most recent available visit where a given test was assessed, when available. These included global CDR (CDRGLOB), CDR Sum of Boxes (CDRSUM), Mini-Mental State Examination ([MMSE]; NACCMMSE), logical memory immediate recall, (LOGIMEM), logical memory delayed recall (MEMUNITS), digit span forward (DIGIF), digit span backward (DIGIB), Trail Making Test Part A ([TMT-A]; TRAILA), Trail Making Test Part B ([TMT-B]; TRAILB), Wechsler Adult Intelligence Scale Digit Symbol Substitution Test ([WAIS DS]; WAIS), animal list generation/animal fluency (ANIMALS), vegetable list generation/vegetable fluency (VEG), and Boston Naming Test, 30 odd items ([BNT]; BOSTON), as previously described (22, 34–37).

### Data analysis

Multivariate logistic regression analysis was performed with MedCalc (MedCalc Software Ltd, Ostend, Belgium). All other statistical analyses were performed with GraphPad Prism version 9 (GraphPad Software, Inc., La Jolla, CA). Data were normalized for heatmaps by subtracting the mean of each cognitive/neuropsychological test for each pathologically defined group from the mean of the control group for that variable, then dividing each group by the maximum value for each test to define a range from −1 to 1 for comparison and easy visualization. Proportions of cases with gender, race, *APOE* status, and neuropathologic comorbidities were calculated using the Fisher exact test. Differences between age, education, CDR, CDR sum of boxes, MMSE, and all neuropsychological variables between pathologically-defined groups were evaluated using multiple t-tests and correlated with linear regression modeling using Pearson correlation coefficient. Statistical significance was set at α = 0.05.

## RESULTS

### Demographic, social, and genetic features of LATE-NC and ADNC cohorts

The control group patients were significantly younger than the isolated LATE-NC (76.3 ± 2.8 vs 86.2 ± 2.0 years; p = 0.0021), isolated ADNC (79.8 ± 0.5 years; p = 0.0106), and concurrent LATE-NC/ADNC subjects (83.4 ± 0.6 years; p < 0.0001). There were no significant differences between the 4 groups regarding gender or years of education, however, the isolated ADNC and concurrent LATE-NC/ADNC groups had a higher proportion of Caucasian subjects compared to the control cohort. The isolated ADNC-only and concurrent LATE-NC/ADNC groups had a significantly lower proportion of cases with at least one *APOE* ε2 allele compared to the control group (p = 0.0316 and p = 0.0154, respectively) and a significantly higher proportion of cases with at least one *APOE* ε4 allele (p = 0.0003 and p < 0.0001, respectively) (Table).

### Cognitive and neuropsychological effects of isolated LATE-NC progression

**Figure 1.**
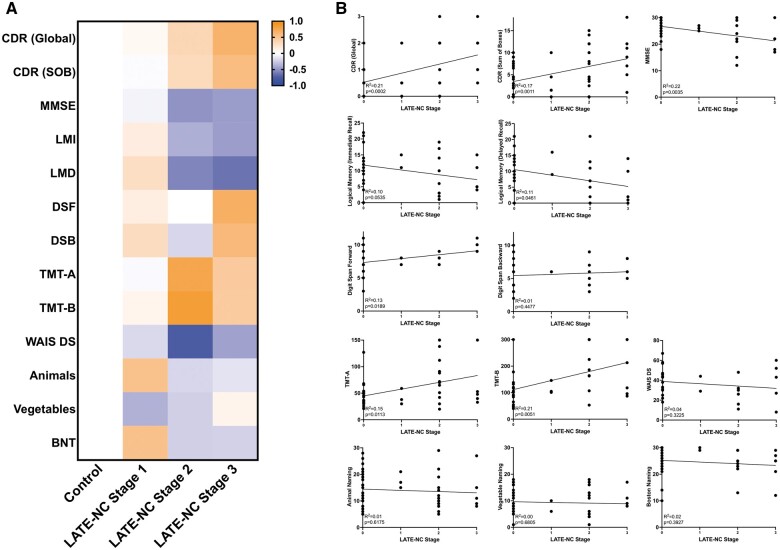
Cognitive and neuropsychological effects of progressive LATE-NC stages. **(A)** Heatmap demonstrating deleterious effects of LATE-NC stages (each cognitive/neuropsychological variable is normalized to a scale of −1 to 1 for easy visualization) and **(B)** linear regression models demonstrating statistically significant progressively deleterious effects of increasing LATE-NC stages for all general cognitive measures and a subset of neuropsychological domains. CDR, clinical dementia rating; MMSE, mini-mental state examination; LMI, logical memory immediate recall; LMD, logical memory delayed memory; DSF, digit span forward; DSB, digit span backward; TMT-A, trail making test A; TMT-B, trail making test B; WAIS DS, Wechsler adult intelligence scale digit symbol substitution; Animals, animal list generation/animal fluency; Vegetables, vegetable list generation/vegetable fluency; BNT, Boston naming test.

LATE-NC restricted only to the amygdala (Stage 1) did not demonstrate a significant deleterious impact on any cognitive or neuropsychological domain. With progression of neuronal TDP-43 inclusions to the hippocampus (LATE-NC Stage 2) there was a significantly deleterious effect on global CDR, CDR sum of boxes, and MMSE, as well as neuropsychological domains involving processing speed/executive function (TMT-A and TMT-B). In LATE-NC Stage 3, global CDR, CDR sum of boxes, and MMSE were significantly impacted compared to the control cohort, and there were nonsignificant trends toward worse logical memory function (immediate and delayed recall), while DSF was actually increased (Fig. 1A and Supplementary Data Table). These same trends in cognitive and neuropsychological testing with isolated LATE-NC stage were demonstrated using linear regression models (Fig. 1B). These results suggest a deleterious effect on global cognition, and some aspects of logical memory, executive function, and processing speed in subjects with isolated LATE-NC, without significant impairment of attention or language.

### Cognitive and neuropsychological effects of isolated ADNC progression

**Figure 2.**
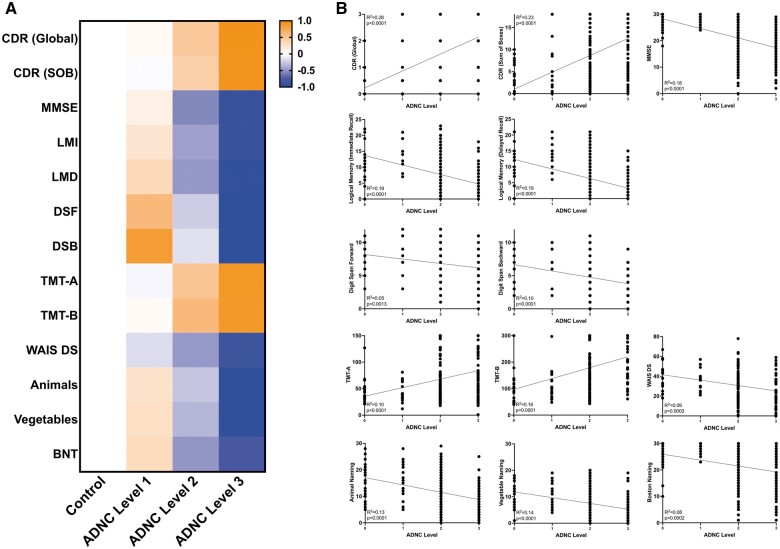
Cognitive and neuropsychological effects of progressive ADNC levels. **(A)** Heatmap demonstrating deleterious effects of ADNC levels (each cognitive/neuropsychological variable is normalized to a scale of −1 to 1 for easy visualization) and **(B)** linear regression models demonstrating statistically significant progressively deleterious effects of increasing ADNC levels for all cognitive and neuropsychological domains. CDR, clinical dementia rating; MMSE, mini-mental state examination; LMI, logical memory immediate recall; LMD, logical memory delayed memory; DSF, digit span forward; DSB, digit span backward; TMT-A, trail making test A; TMT-B, trail making test B; WAIS DS, Wechsler adult intelligence scale digit symbol substitution; Animals, animal list generation/animal fluency; Vegetables, vegetable list generation/vegetable fluency; BNT, Boston naming test.

ADNC groups demonstrated a stepwise deleterious effect in all cognitive and neuropsychological tests according to progressive ADNC levels (Fig. 2A, B). No significant effect on any cognitive and neuropsychological variables was noted in subjects with isolated “low” ADNC (Level 1). Patients with “intermediate” ADNC (Level 2) demonstrated a deleterious pattern on cognition in terms of global CDR, CDR sum of boxes, MMSE, immediate and delayed recall of logical memory, Boston Naming Test, TMT-A, TMT-B, and WAIS digit substitution score, but attention-related variables and other language-related testing were relatively spared. “High” level ADNC (Level 3) demonstrated a more severe deleterious effect across all cognitive and neuropsychological domains compared to both controls and subjects with lower levels of ADNC. Notably, subjects with isolated ADNC had worse performance across all cognitive and neuropsychological tests compared to subjects with isolated LATE-NC.

### Cognitive and neuropsychological effects of comorbid LATE-NC and ADNC

**Figure 3.**
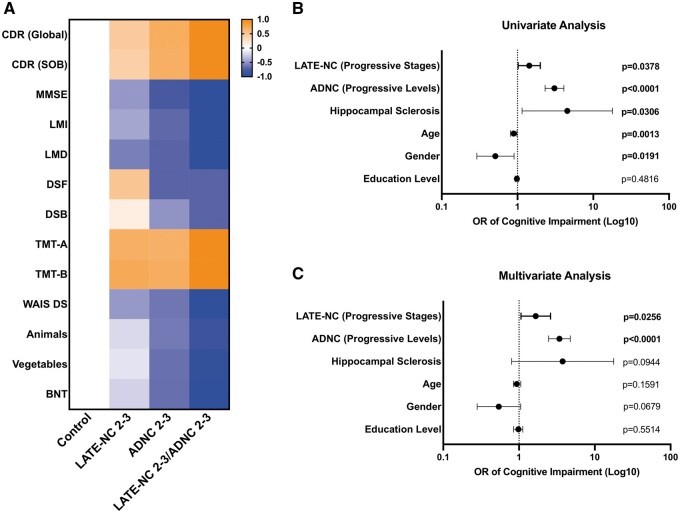
Cognitive and neuropsychological effects of concurrent LATE-NC and ADNC. **(A)** Heatmap demonstrating effects of combinations of LATE-NC stages and ADNC levels on cognitive and neuropsychological domains and **(B)** univariate and **(C)** multivariate logistic regression model-derived odds ratios (OR) and 95% confidence intervals (CI) of the presence of LATE-NC stage, ADNC level, hippocampal sclerosis, age, gender, and education level on the development of cognitive impairment (CDR≥0.5) (n = 475). CDR, clinical dementia rating; MMSE, mini-mental state examination; LMI, logical memory immediate recall; LMD, logical memory delayed memory; DSF, digit span forward; DSB, digit span backward; TMT-A, trail making test A; TMT-B, trail making test B; WAIS DS, Wechsler adult intelligence scale digit symbol substitution; Animals, animal list generation/animal fluency; Vegetables, vegetable list generation/vegetable fluency; BNT, Boston naming test.

The presence of concurrent LATE-NC Stages 2–3 and ADNC Levels 2–3 resulted in the worst cognitive function across all cognitive and neuropsychological testing (Fig. 3A), as demonstrated in previous studies (9, 11, 16–18). There was a trend toward worse cognitive and neuropsychological test scores in all domains in subjects with concurrent LATE-NC Stages 2–3 and ADNC Stages 2–3 compared to isolated LATE-NC Stages 2–3 or isolated ADNC Levels 2–3. In addition, the presence of concurrent LATE-NC Stage 1/ADNC Level 1 demonstrated a relatively small but statistically significant impairment in global CDR, despite no significant effect of either LATE-NC Stage 1 or ADNC Level 1 in isolation. There was no clinically significant impact on any additional neurocognitive variables in concurrent LATE-NC Stage 1/ADNC Level 1 subjects, although this may be partly due to the low sample size (Supplementary Data Table). Multivariate logistic regression modeling demonstrated a significant independent association of progressive LATE-NC stage and ADNC level with cognitive impairment (defined using a threshold of either CDR ≥0.5 or ≥1) at a subject’s final cognitive evaluation (n = 475); each LATE-NC stage had an odds ratio (OR) of 1.66 (95% confidence interval [CI] 1.06–2.60; p = 0.0256) and ADNC had an OR of 3.41 (95% CI 2.46–4.74; p < 0.0001) for cognitive impairment, with the subject’s age, sex, years of education, and presence/absence of hippocampal sclerosis used as covariates (Fig. 3B, C).

**Table.** Demographic and genetic data in control, LATE-NC, ADNC, and concurrent LATE-NC/ADNC subjects

	Control	LATE-NC only	p-value	ADNC only	p-value	Concurrent LATE-NC/ADNC	p-value
Number	28	31	–	244	–	172	–
Mean age (years)	76.3 ± 2.8	86.0 ± 2.2	**0.0028**	79.8 ± 0.5	**0.0106**	83.4 ± 0.6	**<0.0001**
Gender (M:F)	13:15	15:16	0.8804	135:109	0.3705	82:86	0.2334
Race							
Caucasian	85.7%	93.3%		94.3%		92.9%	
African American/Black	3.6%	6.5%		4.1%		5.4%	
Asian	10.7%	0%		0.9%		1.2%	
Other	0%	3.2%	0.2123	0.9%	**0.0032**	0.6%	**0.0295**
Education (years)	16.2 ± 0.5	16.9 ± 0.5	0.2699	16.0 ± 0.2	0.7451	16.1 ± 0.2	0.8507
*APOE* Status							
≥1 *APOE* ε2 allele	24.0%	20.7%	0.2918	8.6%	**0.0316**	7.1%	**0.0154**
≥1 *APOE* ε4 allele	16.0%	20.7%	0.6582	50.0%	**0.0003**	56.5%	**<0.0001**
Hippocampal sclerosis	0%	48.4%	**<0.0001**	5.7%	0.1931	39.3%	**<0.0001**

Bold text indicates significance at a level of <0.05; *APOE* status was not available for all cases.

### Frequency of hippocampal sclerosis in LATE-NC, ADNC, and combined pathology subjects

Hippocampal sclerosis was not seen in any of the control group cases but was found much more frequently in the isolated LATE-NC cohort as a whole (48.4%; p < 0.0001), and the concurrent LATE-NC/ADNC cohort (39.3%; p < 0.0001) (Table). Hippocampal sclerosis (HS) was not seen in any LATE-NC Stage 1 or “low” level ADNC (Level 1) subjects. HS was present in 52.6% of isolated LATE-NC Stage 2 (p < 0.0001) and 71.4% of isolated LATE-NC Stage 3 (p < 0.0001), whereas only 7.5% of isolated ADNC Level 2 (p = 0.1358) and 5.0% of isolated ADNC Level 3 (p = 0.2271) had HS, consistent with the known association between LATE-NC and hippocampal sclerosis. Twenty-five percent (1 in 4 cases) of concurrent LATE-NC Stage 1/ADNC Level 1 had unilateral hippocampal sclerosis, significantly greater than the control cohort (p = 0.0163), suggesting an association between concurrent disease (even with low disease burden) and the higher incidence of hippocampal sclerosis (although this may be confounded by difficulty in identifying neuronal cytoplasmic TDP-43 inclusions in the CA1 subregion in cases with hippocampal sclerosis, so there is a possibility that the case with hippocampal sclerosis may be incorrectly classified as LATE-NC Stage 1 instead of LATE-NC Stage 2). Regarding the cohort with concurrent LATE-NC Stages 2–3/ADNC Levels 2–3, 39.3% had hippocampal sclerosis, significantly greater than the control (p < 0.0001) and ADNC cohorts (p < 0.0001), but not significantly different from isolated LATE-NC Stages 2–3 (p = 0.1135).

## DISCUSSION

LATE-NC is a recently codified neuropathologic entity that has grown out of the previously morphologically defined “hippocampal sclerosis of aging.” Some degree of LATE-NC is a frequent finding in autopsy cohorts of elderly individuals and may be concurrent with other common neurodegenerative diseases, including Alzheimer disease, LBD, and cerebrovascular disease, or more rarely as an isolated finding (1, 9–12, 14). Clinically, patients with isolated LATE-NC are more likely to initially present with more normal cognition and develop symptoms more slowly or at an older age than subjects with ADNC (1, 4, 16, 18, 38, 39), although LATE-NC has also been associated with increased impairment of cognition in subjects with ADNC, LBD, PART, and other neurodegenerative entities (1, 8, 10, 11, 13, 14, 16, 22).

Given that LATE-NC shares some similar morphologic features and a common protein inclusion (TDP-43) with other neurodegenerative diseases, including FTLD-TDP (types A–E) and ALS, there remains debate as to the nature of LATE-NC and its relationship to these other entities (5, 8, 40, 41). While no accepted set of criteria for the definitive distinction between LATE-NC and FTLD-TDP yet exists, there have been recent recommendations for differentiating between the 2 disorders from a neuropathologic standpoint (8). TDP-43 in LATE-NC may be similar to FTLD-TDP types A and B, however, it is histomorphologically distinct from types C–E. Moreover, the presence of known pathogenic mutations associated with FTLD-TDP (such as C9orf72 and *GRN*) are incompatible with a diagnosis of LATE-NC, and an earlier age of onset, high burden of middle frontal gyrus TDP-43 immunoreactive inclusions, and TDP-43 inclusions in subcortical structures, the brainstem, or spinal cord should favor a diagnosis of FTLD-TDP and/or ALS. The LATE-NC subjects included in this study were significantly older (p < 0.0001) and had better overall cognition at their final clinical encounter (p < 0.0001) than NACC subjects with FTLD-TDP.

In this study, we demonstrate impairment of global cognition and some measurements of logical memory, processing speed, and executive function with progressive LATE-NC stages (Fig. 1), which follows a similar, if less severe, trajectory with progressive ADNC levels (Fig. 2). Similar to previous studies, we found that the combination of ADNC and LATE-NC resulted in further impairment of many of the cognitive and neuropsychological domains assessed. While combined low levels of LATE-NC (Stage 1) and ADNC (Level 1) only caused small impairments detectable in global CDR, the combination of symptomatic LATE-NC (Stages 2–3) and symptomatic ADNC (Levels 2–3) caused additional impairment in global cognitive measures and a number of the more specific neuropsychological tests. Importantly, LATE-NC stage and ADNC level were each independently associated with cognitive impairment in multivariate logistic regression models; LATE-NC stage appears to contribute less to cognitive impairment than does ADNC level, but there is a significant increase in the likelihood of cognitive impairment with increasing LATE-NC stages and LATE-NC has a clear additive effect when concurrent with ADNC (Fig. 3). The incidence of hippocampal sclerosis was increased in LATE-NC Stages 2–3 compared to controls and LATE-NC restricted to the amygdala, although no significant difference was found between Stages 2 and 3. This is in contrast to isolated ADNC (without concurrent LATE-NC) which does not appear to significantly increase the incidence of HS compared to controls, although there is an increased frequency of HS in combined low-level LATE-NC/ADNC pathology compared to controls or either of these low-level findings in isolation. The odds ratio for cognitive impairment in subjects with hippocampal sclerosis was significant in univariate analysis, but no significant effect on cognition was identified when LATE-NC was introduced as a covariate (Fig. 3B, C). These data are congruent with previous findings suggesting that HS is a frequent feature of LATE-NC and that the majority of HS cases have underlying LATE-NC pathology (41–45). HS has a significantly deleterious effect on cognition, however, in the current cohort, this effect appears to primarily be in the context of TDP-43 immunoreactive inclusions in the hippocampus and/or middle frontal gyrus.

There are several limitations associated with this study. The first is inherent to the NACC dataset itself, which is likely not representative of the general population. This cohort is enriched for non-Hispanic Caucasian patients, patients of higher socioeconomic status, patients with higher education, patients with rare neurodegenerative diseases and more severe neuropathologic findings and clinical symptoms, and patients with more frequent *APOE* ε4 alleles compared to community-based autopsy cohorts. In addition, there are relatively few cases that could be considered “controls” by either cognitive or neuropathologic measures. With regard to LATE-NC and ADNC staging, autopsy data on TDP-43 inclusions and Thal phase were not included in the NACC NP data set until version 10 in 2014 (16), and there is currently no specific “LATE-NC” designation. Therefore, the majority of cases in the NACC dataset could not be assigned a LATE-NC stage and many could not be assigned an official ADNC level. We included only cases with enough neuropathologic information available to assign these scores, which accounted for our relatively low number of subjects compared to the full NACC cohort, particularly in the isolated LATE-NC groups. This makes it impossible to draw meaningful conclusions about the prevalence of LATE-NC in this cohort, may result in some comparisons being underpowered, and may explain why some neuropsychological tests were significant in LATE-NC Stage 2 but not LATE-NC Stage 3. Future research into LATE-NC stage subgroups with larger cohorts should further elucidate differences in neuropsychological profiles in these groups. Additionally, the neuropathologic data available from NACC assess the distribution of pathology in the brain but not the severity of pathology within a brain region, which may be an important factor in predicting cognitive status in some diseases, particularly those with low overall disease burden or occurring at early stages (46–50). By necessity, this was a cross-sectional study and as such could not directly measure the cognitive or neuropsychological impact of progressive LATE-NC or ADNC, however, the development of in vivo biomarkers may allow for longitudinal assessment of cognitive function in individuals with isolated and mixed neurodegenerative pathologies in the near future (3).

Overall, our findings suggest that LATE-NC has significant effects on global cognition and select neuropsychological domains as TDP-43 progresses to the hippocampus (Stage 2) and frontal neocortex (Stage 3). LATE-NC also further impairs cognitive function in subjects with concomitant ADNC, and multivariate logistic regression analysis suggests that these 2 disease processes have independent deleterious effects on cognition. These data add to the existing knowledge on the clinical consequences of LATE-NC and the growing literature on the effects of multiple concurrent neurodegenerative pathologies. Given the prevalence of these 2 entities and the frequency of their co-occurrence and with additional pathologies and the current need for developing biomarkers and targeting therapies towards individual protein accumulations and neuronal populations, determining the relative effects of each disease process is critical for clinical assessment and drug development.

## Supplementary Material

nlad098_Supplementary_Data
