# Supplementary material for: Cognitive symptoms progress with limbic-predominant age-related TDP-43 encephalopathy stage and co-occurrence with Alzheimer disease
Source: J Neuropathol Exp Neurol. 2023 Nov 15;83(1):2–10. doi: 10.1093/jnen/nlad098 (PMC10746699; doi:10.1093/jnen/nlad098)
Supplement: nlad098_Supplementary_Data [file nlad098_supplementary_data.zip › nlad098_Supplementary_Data/Supplemental Table.pdf]

Supplemental Table. Cognitive and neuropsychological effects of progressive LATE stages, ADNC levels, and combined LATE/ADNC pathology.

	**Clinical Dementia Rating (CDR)**		**MMSE**	**Memory**		**Attention**		**Processing Speed**		**Executive**	**Language**		
	Global	Sum of Boxes		Logical Memory Immediate Recall	Logical Memory Delayed Recall	Digit Span Forward	Digit Span Backward	TMT-A	WAIS DS	TMT-B	Animals	Vegetables	BNT
**Control**	0.6 ± 0.1	3.7 ± 0.6	26.7 ± 0.6	11.7 ± 1.0	10.3 ± 1.1	7.4 ± 0.4	5.4 ± 0.5	43.8 ± 4.4	39.8 ± 2.6	106.6 ± 11.7	14.3 ± 1.3	9.9 ± 0.9	25.0 ± 1.0
**LATE Stage 1**	0.7 ± 0.3	3.5 ± 1.8	26.0 ± 0.4	13.0 ± 1.3	12.5 ± 2.2	7.7 ± 0.3	6.0 ± 0.0	42.3 ± 6.7	36.5 ± 4.7	117.3 ± 11.1	17.7 ± 1.4	8.0 ± 1.3	29.5 ± 0.3
p-value	0.5790	0.9245	0.7180	0.7350	0.6148	0.8538	0.7055	0.9212	0.7542	0.7747	0.4232	0.5970	0.2505
**LATE Stage 2**	1.2 ± 0.2	6.9 ± 1.2	21.4 ± 1.4	8.6 ± 1.4	6.0 ± 1.1	7.4 ± 0.2	5.0 ± 0.3	79.3 ± 10.4	26.4 ± 1.9	212.2 ± 20.7	13.0 ± 1.6	8.7 ± 1.4	23.0 ± 1.2
p-value	**0.0069**	**0.0128**	**0.0094**	0.2130	0.1019	0.9843	0.6588	**0.0047**	0.0654	**0.0007**	0.5705	0.5196	0.4036
**LATE Stage 3**	1.6 ± 0.3	9.0 ± 1.9	21.8 ± 1.6	8.0 ± 1.7	5.4 ± 2.2	9.8 ± 0.3	6.6 ± 0.5	64.8 ± 17.1	34.0 ± 7.3	162.8 ± 32.4	13.4 ± 2.9	10.4 ± 1.4	23.1 ± 2.1
p-value	**<0.0001**	**0.0024**	**0.0093**	0.1063	0.1211	**0.0195**	0.3285	0.1649	0.5279	0.1248	0.8033	0.8202	0.4915
**ADNC Level 1**	0.7 ± 0.2	3.5 ± 1.2	28.1 ± 0.4	13.5 ± 0.9	12.9 ± 0.9	8.6 ± 0.6	7.1 ± 0.5	41.6 ± 3.6	37.6 ± 2.4	112.0 ± 12.2	15.9 ± 1.5	11.3 ± 1.0	27.6 ± 0.5
p-value	0.5901	0.8891	0.1066	0.2646	0.1335	0.0961	0.0898	0.6271	0.8489	0.7951	0.4119	0.2786	0.0597
**ADNC Level 2**	1.4 ± 0.1	7.8 ± 0.5	20.9 ± 0.6	8.1 ± 0.5	6.6 ± 0.5	6.9 ± 0.2	5.1 ± 0.2	66.8 ± 3.0	31.3 ± 1.2	182.5 ± 6.4	12.5 ± 0.5	8.1 ± 0.4	20.9 ± 0.6
p-value	**<0.0001**	**0.0010**	**0.0016**	**0.0271**	**0.0245**	0.4180	0.5996	**0.0147**	**0.0420**	**0.0008**	0.2725	0.1296	**0.0376**
**ADNC Level 3**	2.1 ± 0.1	12.5 0.4	17.8 ± 0.4	4.6 ± 0.3	3.4 ± 0.3	6.1 ± 0.2	3.4 ± 0.1	85.5 ± 3.4	24.5 ± 1.1	217.3 ± 5.6	7.9 ± 0.4	4.8 ± 0.3	18.4 ± 0.5
p-value	**<0.0001**	**<0.0001**	**<0.0001**	**<0.0001**	**<0.0001**	**0.0420**	**0.0008**	**0.0003**	**0.0015**	**<0.0001**	**<0.0001**	**<0.0001**	**0.0033**
**LATE Stage 1/ADNC Level 1**	1.3 ± 0.7	6.8 ± 3.8	27.3 ± 0.3	8.0 ± 1.8	6.7 ± 2.5	6.3 ± 0.6	5.7 ± 0.3	37.0 ± 3.1	41.3 ± 1.5	107.3 ± 9.6	16.6 ± 0.3	9.7 ± 1.3	27.6 ± 1.0
p-value	**0.0231**	0.1423	0.7692	0.2748	0.3369	0.3686	0.8800	0.6323	0.8554	0.9841	0.5688	0.9439	0.3991
**LATE Stage 2-3**	1.3 ± 0.2	7.5 ± 1.0	22.1 ± 1.1	8.3 ± 1.0	5.8 ± 1.0	8.4 ± 0.3	5.7 ± 0.3	75.5 ± 8.7	31.3 ± 3.2	197.6 ± 17.5	13.1 ± 1.3	9.2 ± 1.1	23.1 ± 1.0
p-value	**0.0007**	**0.0024**	**0.0054**	0.0974	**0.0381**	0.1642	0.7833	**0.0080**	0.1917	**0.0016**	0.5731	0.6476	0.3281
**ADNC Level 2-3**	1.8 ± 0.1	10.6 ± 0.3	18.4 ± 0.4	6.2 ± 0.3	4.9 ± 0.3	6.0 ± 0.1	4.4 ± 0.1	74.8 ± 2.2	28.7 ± 0.8	194.5 ± 4.3	10.4 ± 0.3	6.6 ± 0.3	19.5 ± 0.4
p-value	**<0.0001**	**<0.0001**	**0.0001**	**0.0016**	**0.0018**	**0.0246**	**0.0182**	**<0.0001**	**0.0001**	**<0.0001**	**0.0194**	**0.0087**	**0.0265**
**LATE Stage 2-3/ADNC Level 2-3**	2.2 ± 0.1	13.7 ± 0.3	16.1 ± 0.5	3.6 ± 0.3	2.6 ± 0.3	6.0 ± 0.2	4.0 ± 0.2	90.0 ± 3.0	21.9 ± 1.0	231.9 ± 5.7	8.8 ± 0.4	5.1 ± 0.3	16.6 ± 0.6
p-value	**<0.0001**	**<0.0001**	**<0.0001**	**<0.0001**	**<0.0001**	**0.0138**	**0.0316**	**<0.0001**	**0.0001**	**<0.0001**	**0.0003**	**<0.0001**	**0.0002**

Note: bold text indicates significance at a level of <0.05; Not all neuropsychological variables were available for all cases
