# Supplementary figures and images for: Cognitive symptoms progress with limbic-predominant age-related TDP-43 encephalopathy stage and co-occurrence with Alzheimer disease
Source: J Neuropathol Exp Neurol. 2023 Nov 15;83(1):2–10. doi: 10.1093/jnen/nlad098 (PMC10746699; doi:10.1093/jnen/nlad098)

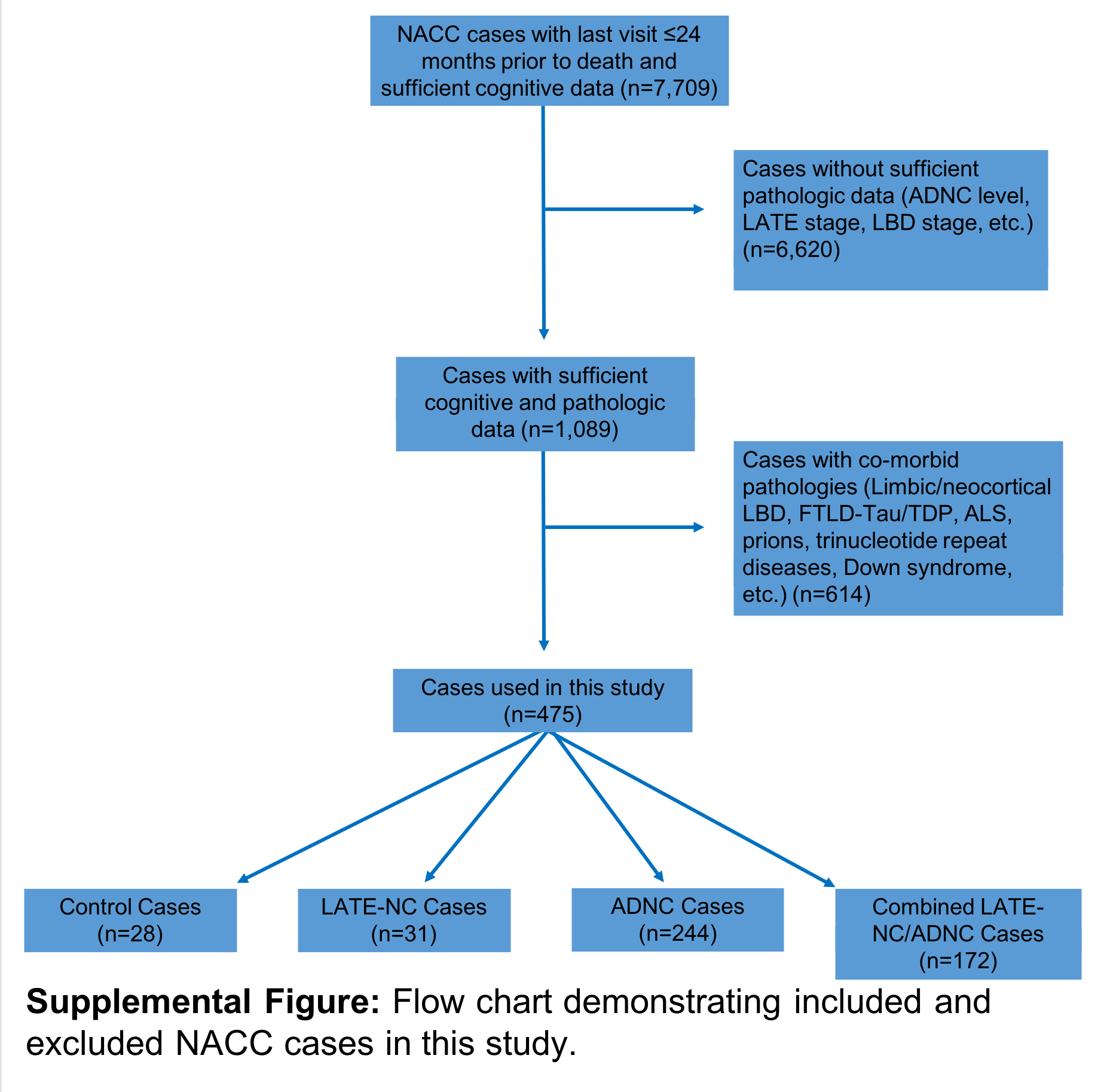

Supplement: nlad098_Supplementary_Data [file nlad098_supplementary_data.zip › nlad098_Supplementary_Data/Supplemental Figure.tiff]
